# New avenues for matter-wave-enhanced spectroscopy

**DOI:** 10.1007/s00340-016-6573-y

**Published:** 2016-12-09

**Authors:** Jonas Rodewald, Philipp Haslinger, Nadine Dörre, Benjamin A. Stickler, Armin Shayeghi, Klaus Hornberger, Markus Arndt

**Affiliations:** 1 Faculty of Physics, VCQ, University of Vienna, Boltzmanngasse 5, 1090 Vienna, Austria; 2 University of California, Berkeley, Leconte/Birge Hall, Berkeley, CA 94720 USA; 3 Faculty of Physics, University of Duisburg-Essen, Lotharstraße 1-21, Duisburg, Germany

## Abstract

We present matter-wave interferometry as a tool to advance spectroscopy for a wide class of nanoparticles, clusters and molecules. The high sensitivity of de Broglie interference fringes to external perturbations enables measurements in the limit of an individual particle absorbing only a single photon on average, or even no photon at all. The method allows one to extract structural and electronic information from the loss of the interference contrast. It is minimally invasive and works even for dilute ensembles.

## Introduction

Our contribution to this special issue is dedicated to Theodor W. Hänsch, who has inspired generations of physicists as a role model for scientific creativity, genius and passion for precision. Seeing how many methods in laser physics, atomic and molecular physics, quantum optics, and high-level spectroscopy Ted Hänsch advanced to unprecedented precision, we are reminded of a remark by Whitehead about philosophy: *The safest general characterization of the European philosophical tradition is that it consists of a series of footnotes to Plato.* [1]. In that spirit, we offer here a ‘footnote’ to Hänsch’s work on spectroscopy and matter-wave interferometry.

In the following, we will focus on prospects for measurements in *OTIMA* [2, 3], an Optical TIme-domain near-field MAtter-wave interferometer for clusters and molecules with pulsed photo-depletion gratings. However, our arguments can be readily transferred to other interferometers for atoms, clusters, or macromolecules that use combinations of mechanical and optical gratings, operating in the matter-wave near-field or far-field, in position space or in the time domain [4, 5].

## Matter-wave interferometry with pulsed photo-depletion gratings

Near-field matter-wave interferometry is based on the discovery of coherent self-imaging behind periodic structures by Talbot [6] and Lau [7]: When a transmission grating of period *d* is illuminated by a plane wave of wavelength $\lambda$, an image of the mask will be reproduced at multiples of the Talbot distance $L_{\mathrm{T}}=d^2/\lambda$ behind the grating—without the need of any focusing optics, simply by virtue of near-field interference. The trick works even for spatially incoherent sources if another grating is inserted before the diffraction mask, again at multiples of the Talbot length. This concept was realized for light [8], X-rays [9, 10] and atoms [11, 12]—also in the time domain [13–15]. Throughout the last decade, Talbot–Lau interferometry has been extended to organic molecules, clusters and biomolecules [3, 16–18].

**Fig. 1:**
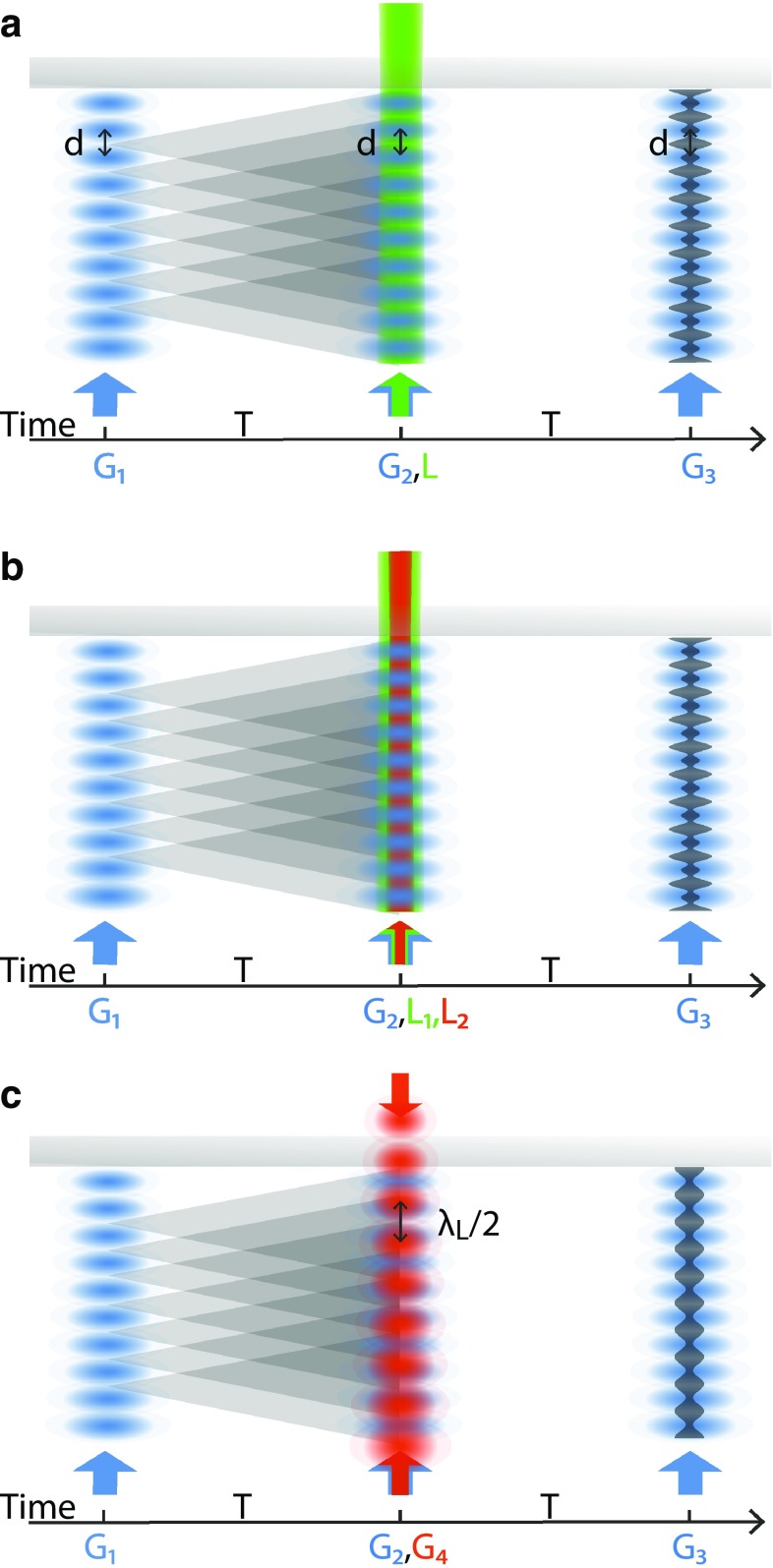
**a** UV–VIS spectroscopy in OTIMA: absorption of a single photon from a running laser wave imparts a recoil to the absorbing cluster or molecule. If the wavelength of the light is comparable to the semiclassical path separation of the delocalized particle, the interference fringe pattern experiences a measurable dephasing (Sect. 3) [19, 20]. Because of the small grating period (79 nm), *single-color* visible or infrared (VIS/IR) spectroscopy requires the collective momentum transfer of several photons or operation of the matter-wave interferometer in higher Talbot orders. **b** VIS/IR spectroscopy: can also be realized by combining a single (VIS/IR) photon of laser beam ${L}_1$ (*red arrow*) with a single UV photon from beam ${L}_2$ (*green arrow*) which provides the required momentum transfer (Sects. 6 and 7). **c** Polarizability spectroscopy: is the least invasive of all three techniques. The off-resonant dipole interaction with the intense laser field ${G}_4$ deforms the matter-wave front—leading to a loss of fringe contrast even without any photo-absorption. This method may be particularly useful for weakly bound van der Waals clusters (Sect. 8)

*OTIMA*, in particular, is an interferometer that utilizes three pulsed photo-depletion gratings [2, 3, 18] to prepare, diffract and detect beams of complex nanoscale particles (Fig. 1). In our experiments, the gratings are realized as retro-reflected fluorine laser beams, at a vacuum ultraviolet wavelength of $\lambda =157.6$ nm, yielding standing light waves with a period of $d\simeq 79$ nm. In the antinodes of the standing light waves, the molecular beam is depleted by ionization, dissociation, or any other mechanism that renders these molecules invisible to the detector further downstream. This way, the light field acts effectively as a periodic absorptive mask. The high laser photon energy of 7.9 eV allows manipulating a wide range of molecules or clusters in the same machine—largely independent of particle-specific narrow optical resonances.

Three gratings are combined to form a complete Talbot–Lau interferometer: the first grating ${G}_1$ establishes a periodic array of possible molecular locations, close to the nodes of the standing wave. The tight confinement of the wave function around these nodes then imposes a momentum uncertainty which ensures a rapid increase in transverse coherence behind the grating—even for an initially incoherent molecular beam. The second grating is positioned such that the incident molecular coherence extends at least over two nodes or antinodes of ${G}_2$. This way, the propagating molecular wave covers two or more semiclassical paths on the way to the final state at ${G}_3$, further downstream. Resonant near-field interference occurs around multiples of the Talbot time $T_{\mathrm{T}}=d^2 m/h$, corresponding to a Talbot length $L_{\mathrm{T}}=v T_{\mathrm{T}}=d^2/\lambda _{\mathrm{dB}}$, for particles of mass *m*. In time-domain interferometry, all particles within the grating area see the same pulse sequence for the same duration, independent of their own velocity *v*.

The molecular fringe pattern can be visualized in various ways: the third grating ${G}_3$ acts as a spatially resolving mask with a resolution of well below $\lambda /2=79$ nm and a post-ionizing time-of-flight mass spectrometer allows recording all particles transmitted by this mask. If the clusters [3, 18] or nanoparticles [21] in the beam have a broad mass distribution with fixed mass separation, and if they all have the same velocity, as often the case in supersonic beams, they realize a ‘comb’ of de Broglie waves. The particles remain, however, mutually incoherent since they are distinguishable. Recording the mass spectrum then corresponds to reading an interference pattern as a function of mass *m* or wavelength $\lambda _{\mathrm{dB}}$. One may also describe this phenomenon as a wave function rephasing in the time-domain [13], without reference to position and independent of the velocity distribution.

We exploit in particular the resonance in particle transmission behind grating ${G}_3$ as a function of the pulse delay between two subsequent gratings $\tau _{ij}=t({G}_i)-t({G}_j)$. This resonance occurs for a symmetric interferometer timing, $T=\tau _{12}=\tau _{23}$, and we find a rapid decrease in the interference contrast when this balance is skewed by more than $\Delta t= \tau _{23}-\tau _{12}\simeq \tau _{23}/N$, where *N* is the number of grating nodes illuminated by the incident molecular beam [22].

In principle, the matter-wave fringes could also be measured directly by plotting the particle transmission versus the lateral displacement of either grating. However, in our case, all three laser beams are retro-reflected by the same mirror to render the system as insensitive to mechanical vibrations as possible. The fringes are thus not affected by slow tilts or shifts of the mirror. Instead, in OTIMA interferometry, the interference contrast can be extracted from a comparison of the interferometer transmission for the case of resonant (symmetric) and non-resonant (slightly asymmetric) laser pulse delays [3]. For this setting, we here propose a variety of new spectroscopy tools and procedures.

## Matter-wave-enhanced recoil spectroscopy (MERS)

A matter-wave interferometer can be used as a single-photon recoil spectrometer by adding a running laser wave *L* close to the central grating ${G}_2$ (Fig. 1a). Absorption of a single photon then imparts a recoil onto the molecule, without providing ‘which-path information’. Subsequent spontaneous reemission of photons would introduce a random phase and decoherence [23], but most macromolecules dissipate the energy radiationless to many lower-lying electronic and vibrational states [20, 24].

Heating of the internal molecular state does not destroy the center-of-mass coherence [25, 26] as long as the internal and external degrees of freedom remain separable. Wavelets associated with the same internal state remain coherent to each other [24]. Absorption inside a matter-wave interferometer thus creates shifted and unshifted molecular fringe patterns which are correlated with heated and unheated internal states. Even if the shifted and the unshifted fringes cannot be resolved, the loss of the total fringe visibility can be used for spectroscopy with high accuracy [19, 20].

In OTIMA interferometry, the momentum imparted by each VUV grating exceeds the absorption recoil of a 0.3–100 μm spectroscopy photon by a factor up to 300. Visible (VIS) and near-infrared (NIR) spectroscopy will therefore work best in higher Talbot orders, when the grating pulse separation time amounts to about two or three Talbot times and the molecular state is delocalized over two or three periods of ${G}_2$. Probing photons with wavelengths around 270–320 nm are for instance required to study the electronic states of aromatic amino acids and nucleotides, peptides and oligonucleotides. Comparing UV spectra of biomolecules in the gas phase with molecules in solution could later provide valuable information about structural changes in these different environments [27, 28].

## Fluorescence recoil spectroscopy (FRS)

If, contrary to the previous assumptions, absorption is followed by fluorescence, the emitted photon will add a recoil to the molecular motion, whose orientation varies randomly for each molecule. This leads to a reduction of the fringe contrast. One can use this loss of visibility to extract fluorescence quantum yields. When the exciting laser illuminates the molecular beam from the front, the absorption recoil does not blur the interference pattern and the timing of the laser pulse determines when and where the molecule is hit relative to the position and time of the second grating pulse. If the fluorescence wavelength distribution is known, the contrast reduction of the matter-wave interference pattern provides a measure for the product of the absorption cross section and the fluorescence yield. The absorption cross section can be extracted independently at low laser power and with the laser beam oriented parallel to the grating *k*-vector. When as little as 10% of all molecules are excited [20], the absorption measurement is only minimally affected by fluorescence.

## Multi-photon recoil spectroscopy (MPRS)

If the probing laser wavelength exceeds the grating period substantially, a single photon cannot provide the recoil to shift the interference pattern sufficiently far. This is for instance the case for vibrational transitions, driven by near-infrared (NIR) or far-infrared (FIR) photons with wavelengths around 3–100 μm. Multi-photon absorption can then still be a viable option if the cumulated recoil of many absorbed photons has sufficient momentum.

**Fig. 2:**
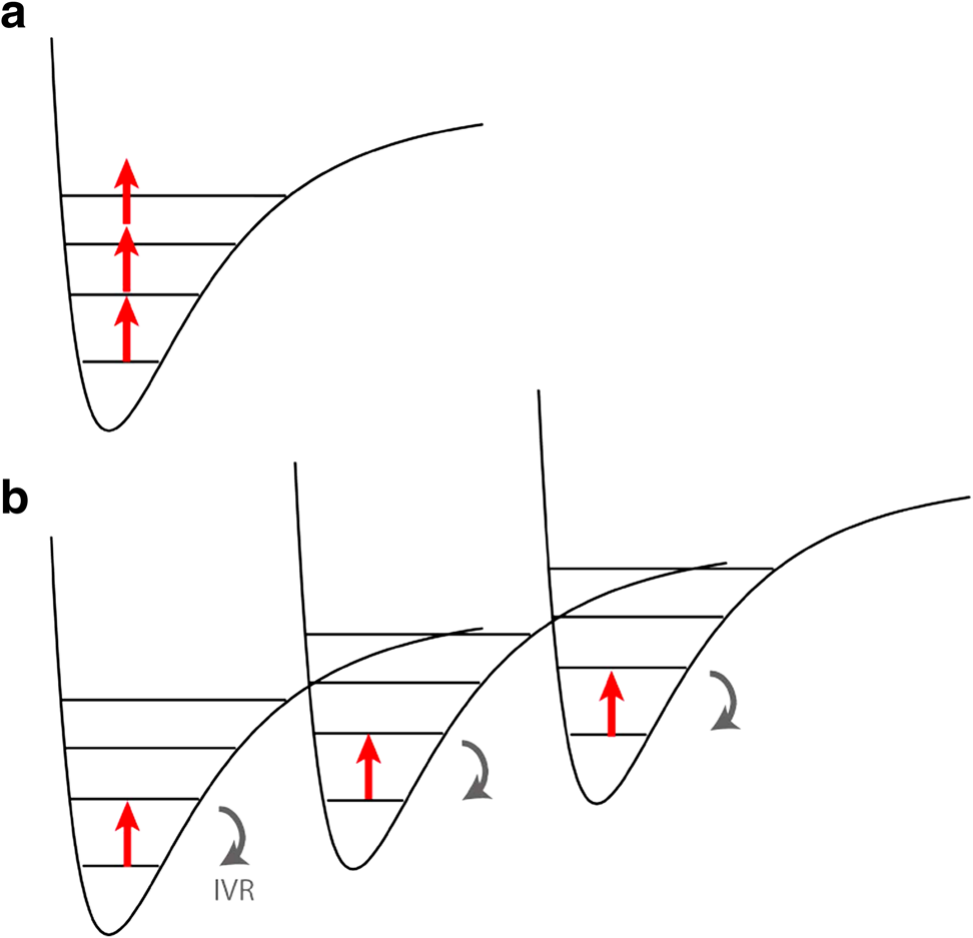
**a** The absorption of multiple photons from a monochromatic source is suppressed due to the anharmonicity bottleneck. **b** Internal vibrational relaxation (IVR) to other modes dissipates the energy and enables the repeated excitation of the same IR transition until sufficient momentum recoil has been accumulated to shift the fringe pattern measurably

*Multi-photon recoil spectroscopy* is conceptually similar to infrared multi-photon dissociation spectroscopy (IR-MPD) [29]. The anharmonicity of molecular potentials usually prevents the subsequent absorption of many monochromatic photons within the same vibrational energy ladder (*anharmonicity bottleneck*, Fig. 2a) [30]. On the other hand, couplings between the vibrational modes can dissipate the absorbed energy (Fig. 2b). In complex particles, vibrational excitations can relax on the picosecond time scale to many vibrational states, i.e., very fast compared to the duration of the nanosecond spectroscopy pulse. Even though multi-photon absorption will lead to internal heating, this is compatible with high-contrast interference as long as it does not provide which-path information by emission of thermal radiation [31]. Sequential absorption with a Poissonian photon number distribution will lead to a biased quantum random walk in momentum. In contrast to the single-photon case, extracting an absolute absorption cross section from the visibility loss is then less direct. However, the spectral line positions and widths will remain measurable.

## Resonance-enhanced multi-photon recoil spectroscopy (REMPRS)

In order to avoid heating and the risk of spectroscopic shifts, conformation changes or even fragmentation, it is desirable to limit the number of photons required to retrieve information—even in the infrared regime. This challenge has been addressed in physical chemistry by *action spectroscopy* where the absorption of a few photons may lead to a detectable ‘action’, for instance the detachment of an additional messenger atom. Action spectroscopy has been very successful in cluster physics [29]. A prominent example is the spectroscopy of impurities in helium nanodroplets where the deposition of 1 eV of energy even suffices to boil off 2000 helium atoms [32]. However, the attached messenger atom or the environment, such as a liquid helium nanodroplet, may also influence the electronic structure of the host molecule [33].

**Fig. 3:**
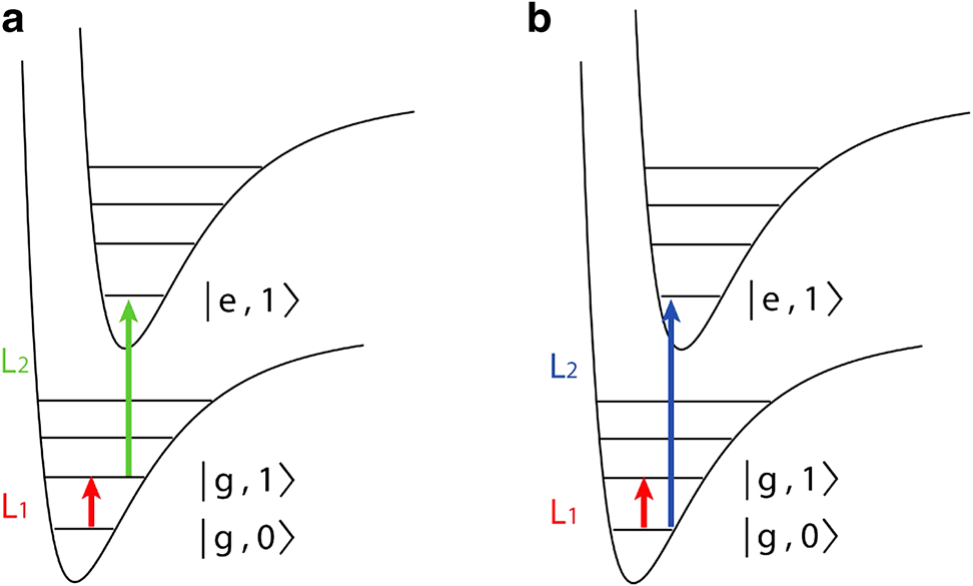
**a** In IR–UV-recoil dip spectroscopy the matter-wave dephasing action of a UV photon is suppressed by emptying the ground state in a resonant IR transition. **b** In double resonant IR–UV-recoil spectroscopy the kick of the UV photon is conditioned on the prior absorption of the IR or VIS photon

We suggest that it is possible to avoid the need for messengers and artificial environments based on a recoil analog of resonance-enhanced multi(two)-photon ionization spectroscopy (REMPI/R2PI) [35]. In matter-wave-enhanced resonant multi-photon recoil spectroscopy *(REMPRS/R2PRS)*, the spectroscopy photon from laser beam ${L}_1$ triggers the absorption of a photon of high momentum from laser beam ${L}_2$. We illustrate the idea in Figs. 1b and 3a where the first photon from laser beam ${L}_1$ excites the molecule for instance from the electronic and vibrational ground state $|g,0\rangle$ to the higher-lying vibrational state $|g,1\rangle$ and a photon from the more energetic laser ${L}_2$ couples this state to the upper electronic state $|e,1\rangle$, imparting the required kick (see Fig. 3a). This method is appealing for particles where photo-ionization has been notoriously difficult and photodissociation channels are not available, as is the case for many massive biomolecules [36–38].

## Matter-wave-enhanced recoil dip spectroscopy (RDS)

While in our previous examples the resonant reduction of matter-wave contrast was assumed to provide the spectroscopic signal, we illustrate in Figs. 1b and 3b how *recoil dip spectroscopy* can even restore and enhance this contrast on resonance. We assume that the absorption of a single (V)UV photon from $|g,0\rangle$ to $|e,1\rangle$ imparts sufficient recoil to reduce the matter-wave visibility. However, we can deplete the ground state $|g,0\rangle$ by coupling it resonantly to a neighboring vibrational state of the same electronic manifold $|g,1\rangle$. This reduces the UV absorption and raises the fringe contrast again. Dip spectroscopy may appear counterintuitive in comparison with earlier results from atom interferometry [34] where an increase in the number of absorbed quanta led to a decrease in fringe contrast. In contrast to that, reemission is suppressed in many molecules during their transit through the interferometer. OTIMA offers a suitable frame for this scheme since the nanosecond precise timing allows depleting the ground state prior to the UV absorption and with a lead time shorter than the life time of the excited state.

*IR–UV dip spectroscopy* requires that the UV photon couples efficiently to one particular vibrational ground state but substantially less to the IR excited vibrational mode. In many small- and medium-sized molecules, it is possible to excite electronic transitions with vibrational resolution. In these cases, recoil dip spectroscopy (RDS) is a realistic option. Even if the UV transitions are broadened when they couple to short-lived excited states, IR dip spectroscopy should provide resolution of the vibrational ground states, as seen in the modulation of the fringe visibility.

*In VIS–UV dip spectroscopy * the transitions couple electronic states and absorption of a visible spectroscopy photon is followed by a UV photon with higher momentum. As before, the method requires that the ground state and the excited state of the electronic transition couple differently to the UV photon.

## Matter-wave-enhanced polarizability spectroscopy (MEPS)

Valuable spectroscopic information can be obtained even without exchanging a single real photon: The atomic or molecular *polarizability* provides important information about the particle composition and structure as well as their van der Waals interactions with molecules or surfaces.

In atom interferometry, the optical polarizability has for instance been measured by imprinting a differential phase on two spatially separated parts of a cloud of ultracold atoms that were then recombined to interfere [39]. Even if the path separation of the matter-wave packets is smaller than the width of the spectroscopy laser beam, they accumulate state-selective phase shifts in the interference pattern, which may provide information about optical polarizabilities [40] or transition dipole matrix elements [41].

This can be generalized to high-mass particles, too. The optical polarizability of complex molecules at fixed wavelength (532 and 157 nm) can be extracted from the diffraction efficiency in the standing light wave in Kapitza–Dirac–Talbot–Lau [42] and OTIMA interferometry [43]. Here, we propose to measure it across a wide spectrum using OTIMA interferometry. By interaction with a tunable *standing light-wave grating* (${G}_4$), close and parallel to ${G}_2$ (see Fig. 1c), the molecular matter-waves acquire a phase shift which reduces their interference contrast.

The effect of the additional grating can be understood in both a classical and a quantum picture: Quantum mechanically, the grating acts like a phase grating, whose period varies with wavelength and whose impact on the matter-wave is a function of the molecular optical polarizability. In a classical picture, the fluctuating array of dipole force microlenses in ${G}_4$ scrambles the molecular interferogram. Tuning the spectroscopy laser then allows one to modulate its fringe contrast (see below).

In contrast to the absorptive spectroscopy, which can be done already with running laser waves, we here rely on the presence of an optical grating to impose strong local dipole forces. They scale with the gradient of the dipole potential and are maximized in a standing light wave. It is favorable if the spectroscopy grating (${G}_4$) phase is unstable since a fluctuating phase ensures that we can ignore residual effects of constructive matter-wave interference that might emerge when the spectroscopy grating ${G}_4$ and the diffraction grating ${G}_2$ have commensurate periods.

## Theoretical description

In order to quantify these statements, we here discuss how the fringe visibility is affected in OTIMA interferometry by the presence of a spectroscopy beam directly after the second grating, ${G}_2$. In general, the interference signal is calculated by combining the effect of each individual grating on the incoming matter wave with its free propagation between the gratings [2, 22, 44].

Exploiting that the transit through each individual laser grating can be described in the eikonal approximation [45], the interaction between the matter wave and grating ${G}_k$, $k = 1,2,3$, is characterized by the eikonal phase shift $\phi _0^{(k)} = 4 \pi E^{(k)} \alpha (\lambda ) / h c \varepsilon _0 A$, and by the mean number of absorbed photons per molecule or cluster, $n_0^{(k)} = 4 E^{(k)} \lambda \sigma _{\mathrm{abs}}(\lambda ) / h c A$ [2]. Here, $E^{(k)}$ is the pulse energy, *A* denotes the laser spot area (flat top assumed), $\alpha (\lambda )$ and $\sigma _{\text{abs}}(\lambda )$ are the molecular polarizability and absorption cross section at the laser wavelength $\lambda$, respectively.

*OTIMA contrast*—In the absence of any additional laser, the sinusoidal visibility of the interferogram can be computed as a function of the laser grating pulse separation time *T* and all known laser parameters

1 $$\begin{aligned} \mathcal{V}_{\mathrm{sin}}&= \frac{2 I_1 (n_0^{(1)}/2) I_1(n_0^{(3)}/2)}{I_0(n_0^{(1)}/2) I_0(n_0^{(2)} / 2) I_0(n_0^{(3)} / 2)} \nonumber \\&\quad\times \left| \frac{\zeta _{\mathrm{coh}} - \zeta _{\mathrm{dep}}}{\zeta _{\mathrm{coh}} + \zeta _{\mathrm{dep}}} J_2 \left( \sqrt{ \zeta ^2_{\mathrm{coh}}- \zeta ^2_{\mathrm{dep}}} \right) \right| , \end{aligned}$$

where $J_n$ and $I_n$ are Bessel functions. The parameter $\zeta _{\mathrm{coh}} = \phi _0^{(2)} \sin ( \pi T / T_{\mathrm{T}})$ describes the coherent evolution induced by the phase grating component in ${G}_2$ and $\zeta _{\mathrm{dep}} = n_0^{(2)}\cos (\pi T / T_{\mathrm{T}})/2$ is related to the photo-depletion of the molecular beam in the anti-nodes of the standing light wave, also in ${G}_2$. The visibility ${\mathcal{V}}_{\mathrm{sin}}$ varies periodically as a function of the pulse separation *T*, and its period is determined by the Talbot time $T_{\mathrm{T}}$.

*Recoil Spectroscopy*—Absorption of photons from a pulsed running wave laser of wavelength $\lambda _{\mathrm{L}}$ in the instant after the second grating pulse will impart a recoil on the absorbing molecule [19]. In practice, one may even overlap ${G}_2$ and the spectroscopy laser on the same spot at the same time using dichroic optics. The resulting reduction of the signal visibility can then be used to extract the absolute absorption cross section of the molecule [20]. Assuming that the probability of absorbing *n* photons is described by a Poisson distribution with mean $n_{\mathrm{L}}(\lambda _{\mathrm{L}}) = \sigma _{\mathrm{abs}}(\lambda _{\mathrm{L}}) E_{\mathrm{L}} \lambda _{\mathrm{L}}/ A_{\mathrm{L}} h c$, the sinusoidal visibility $\widetilde{\mathcal{V}}_{\mathrm{sin}}$ in the presence of the spectroscopy beam can be written as

2 $$\begin{aligned} \frac{\widetilde{\mathcal{V}}_{\mathrm{sin}}}{\mathcal{V}_{\mathrm{sin}}} = \exp \left[ - 2 n_{\mathrm{L}} \sin ^2 \left( \pi \frac{d}{\lambda _{\mathrm{L}}} \frac{T}{T_{\mathrm{T}}} \right) \right] . \end{aligned}$$

Thus, $\ln \widetilde{\mathcal{V}}_{\mathrm{sin}} / \mathcal{V}_{\mathrm{sin}}$ decreases linearly with the product of the total absorption cross section and the recoil laser energy, $\sigma _{\mathrm{abs}}(\lambda _{\mathrm{L}}) E_{\mathrm{L}}$. One can therefore measure the molecular absorption spectrum by varying the laser power at $\lambda _{\mathrm{L}}$ and observing the fringe contrast. This idea can be extended in a straightforward way to recoil dip spectroscopy, where only the readout of the spectrum is modified.

*Polarizability Spectroscopy*—Replacing the running wave laser by a tunable standing light wave grating allows us to measure the molecular polarizability. In this case, the spectroscopy laser acts as a fourth grating with period $\lambda _{\mathrm{L}}/2$. It is timed such that that the free flight to the second grating is negligible. Hence, the interaction between the spectroscopy laser and the molecule is characterized by the eikonal phase $\phi _{\mathrm{L}}(\lambda _{\mathrm{L}}) = 4 \pi E_{\mathrm{L}} \alpha (\lambda _{\mathrm{L}}) / A_{\mathrm{L}} h c \varepsilon _0$ and the mean photon number $n_{\mathrm{L}} (\lambda _{\mathrm{L}}) = 4 E_{\mathrm{L}} \lambda _{\mathrm{L} }\sigma _{\mathrm{abs}}(\lambda _{\mathrm{L}}) / A_{\mathrm{L}} h c$. To avoid moiré-type effects, we propose to induce or maintain phase fluctuations between the spectroscopy grating and the three (phase stable) interferometer gratings. The signal visibility reduction is then

3 $$\begin{aligned} \frac{\widetilde{\mathcal{V}}_{\mathrm{sin}}}{\mathcal{V}_{\mathrm{sin}}} = \frac{\left| J_0 \left[ \sqrt{ \phi _{\mathrm{L}}^2 \sin ^2 \left( \pi \frac{2 d}{\lambda _{\mathrm{L}}} \frac{T}{T_{\mathrm{T}}} \right) - \frac{n_{\mathrm{L}}^2}{4} \cos ^2 \left( \pi \frac{2 d}{\lambda _{\mathrm{L}}} \frac{T}{T_{\mathrm{T}}} \right) } \right] \right| }{I_0 (n_{\mathrm{L}}/2)}. \end{aligned}$$

Varying the laser wavelength $\lambda _{\mathrm{L}}$ in a regime in which photon absorption can be neglected, $n_{\mathrm{L}} \ll 1$, the spectroscopy laser acts as a pure phase grating and the contrast reduction is

4 $$\begin{aligned} \frac{\widetilde{\mathcal{V}}_{\mathrm{sin}}}{\mathcal{V}_{\mathrm{sin}}} = \left| J_0 \left[ \phi _{\mathrm{L}} \sin \left( \pi \frac{2 d}{\lambda _{\mathrm{L}}} \frac{T}{T_{\mathrm{T}} }\right) \right] \right| . \end{aligned}$$

Thus, one can directly extract the spectral molecular polarizability from measuring the contrast reduction for different pulse energies $E_{\mathrm{L}}$.

In deriving the visibility (1), we have neglected additional contrast-reducing processes such as scattering with residual gas atoms [44, 46], thermal decoherence [31] or phase averaging due to machine vibrations or internal molecular dynamics [47, 48]. Such processes would affect the signal visibility with a common pre-factor which cancels in the ratio of the visibility with and without spectroscopy laser. This renders the measurement rather robust with respect to decoherence and dephasing.

## Conclusion

Spectroscopy is an important field of atomic, molecular and optical physics with close ties to areas as diverse as physical and biochemistry, environmental science or laboratory astrophysics. It is therefore important to explore methods which are minimally invasive in the sense that they require the scattering of very few real photons to eventually not even a single one.

Matter-wave interference offers an interesting option as it imposes a very narrow comb of molecular density fringes which serves as a nanoscale ruler, whose position can be read with a sensitivity and accuracy of 10 nm or less.

While a conceptual similarity with classical Moiré shadows is obvious [49], operating in the quantum regime allows one to prepare even narrower fringes and a substantially enhanced sensitivity to fringe displacements. Compared to classical deflectometers, which usually operate with position resolution on the order of tens of micrometers [50, 51], quantum interferometry has the potential of improving the position sensitivity by three to four orders of magnitude. However, substantial future work still needs to be invested in generating sufficiently brilliant molecular beam sources to turn this idea into a generic and universal tool.

Matter-wave-enhanced spectroscopy is promising and useful for isolated molecules and clusters in the gas phase under diverse boundary conditions. It can be beneficial when the absorbed energy is dissipated in internal conversion processes and fluorescence or action spectroscopy fails. This applies to a large class of complex biomolecules and van der Waals clusters.

Interference-assisted absorption spectroscopy is also expected to be favorable for many gas phase neutral vitamins, peptides and proteins with a low vapor pressure, forming only very dilute molecular beams. While interferometry can operate eventually even with a single molecule per shot, direct absorption using Beer’s law would require beam densities many orders of magnitude higher.

Matter-wave interferometry-assisted two-photon and polarizability spectroscopy is also favored over fluorescence methods, where one would usually want to scatter many photons per particle. Multi-photon scattering may lead to excessive heating, particle dissociation or modification. This is the case for weakly bound van der Waals clusters, whose quantum wave nature has been successfully demonstrated in OTIMA interferometry [3, 18].
